# Impact of Frailty in Patients With Continuous‐Flow Left Ventricular Assist Device Therapy in End‐Stage Heart Failure: A Systematic Review and Meta‐Analysis

**DOI:** 10.1111/aor.14998

**Published:** 2025-03-14

**Authors:** Christos Costa, Arian Arjomandi Rad, Yi Ting Yu, Nithiananthan Mayooran, Andrew Xanthopoulos, Marinos Koulouroudias, Robert Vardanyan, Gustavo Antonio Guida, Lydia Wilkinson, Jan Schmitto, Arjang Ruhparwar, Alina Zubarevich, Alexander Weymann, Peyman Sardari Nia, Antonios Kourliouros, Thanos Athanasiou

**Affiliations:** ^1^ Department of Surgery and Cancer Imperial College London London UK; ^2^ Department of Cardiothoracic Surgery John Radcliffe Hospital, Oxford University Hospital NHS Trust Oxford UK; ^3^ Department of Cardiothoracic Surgery Maastricht University Medical Centre Maastricht the Netherlands; ^4^ Barts and the London School of Medicine and Dentistry, Queen Mary University of London London UK; ^5^ Department of Cardiothoracic Surgery King's College Hospital London UK; ^6^ Department of Cardiology University of Thessaly Larissa Greece; ^7^ Department of Cardiothoracic Surgery Bristol Heart Institute Bristol UK; ^8^ Department of Cardiothoracic, Transplant and Vascular Surgery Hannover Medical School Hannover Germany

**Keywords:** Feart Failure, Frailty, LVAD, MCS

## Abstract

**Background:**

Frailty, marked by increased vulnerability and reduced physiological reserve, is common in end‐stage heart failure patients. Continuous flow left ventricular assist devices (LVADs) have improved outcomes, but the impact of frailty on these outcomes is unclear. This systematic review and meta‐analysis investigate the effect of frailty on clinical outcomes in patients undergoing LVAD therapy.

**Methods:**

Following PRISMA guidelines, we searched PubMed, Cochrane, EMBASE, MEDLINE, and Google Scholar up to September 2023 for studies comparing frail and non‐frail patients undergoing LVAD implantation. Data on mortality, hospital length of stay, intubation duration, bleeding, infection, and readmission rates were extracted and analyzed using the Mantel–Haenszel random‐effects model, with heterogeneity assessed by the I2 statistic.

**Results:**

Fifteen studies involving 3458 patients were included. Frailty was significantly associated with higher long‐term mortality (OR: 2.12; 95% CI: 1.17–3.83; *p* = 0.01), but not with short‐term mortality (OR: 1.61; 95% CI: 0.71–3.65; *p* = 0.26), hospital length of stay (MD: 1.93; 95% CI: −9.83 to 13.68; *p* = 0.75), or intubation duration (MD: 34.28; 95% CI: −1.15–69.71; *p* = 0.06). No significant differences were found in bleeding (OR: 1.76; 95% CI: 0.76–4.10; *p* = 0.19), infection (OR: 0.44; 95% CI: 0.11–1.84; *p* = 0.26), or readmission rates (OR: 1.07; 95% CI: 0.78–1.46; *p* = 0.68).

**Conclusion:**

Frail patients with LVADs have higher long‐term mortality but similar short‐term outcomes, hospital stays, intubation times, bleeding, infection, and readmission rates compared to non‐frail patients. These findings highlight the need for tailored strategies to improve outcomes in frail LVAD patients and suggest further research on frailty interventions.

## Graphical abstract

Frailty significantly impacts clinical outcomes in LVAD recipients. This systematic review and meta‐analysis reveal that frail patients experience higher long‐term mortality but comparable short‐term mortality, hospital stay, and complication rates to non‐frail patients. These findings emphasize the importance of tailored interventions to optimize outcomes in frail LVAD recipients.

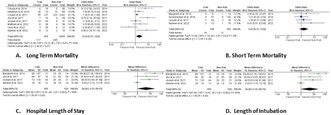

## 1 Background

In recent years, a discernible increase in the number of patients opting for left ventricular assist device (LVAD) implantation has been observed, particularly for destination therapy among individuals ineligible for heart transplantation [1, 2]. The adoption of this therapeutic approach has resulted in enhanced survival rates and various short‐ and long‐term clinical outcomes. Given the heterogeneity in patient characteristics, it becomes imperative to stratify risk within the advanced heart failure population to identify those who are most likely to derive optimal benefits from LVAD therapy.

Frailty manifests as a complex clinical state characterized by heightened physiological vulnerability, diminished stress tolerance, and a gradual decline in physiological functions [3]. One of the most recognized indicators of frailty is Fried's Frailty Index—first proposed in 2001 by Dr. Linda P. Fried. The Fried Frailty Index utilizes five distinct criteria. These criteria include unintentional weight loss, self‐reported exhaustion, weakness as measured by grip strength, slow walking speed, and low physical activity levels. Frailty, according to the Fried Frailty Index, is identified when an individual exhibits three or more of these criteria. This index serves as an indicator of reduced physiological reserve and heightened vulnerability to adverse health outcomes, particularly in the context of the aging population [4].

Existing literature indicates a correlation between frailty and heightened mortality rates in cardiac patients undergoing valve replacements and LVAD implantations. Previous systematic reviews have indicated that frailty is associated with significantly prolonged extubation times, extended hospitalizations, and increased long‐term mortality in advanced heart failure patients who have undergone LVAD implantation [5].

In this study, we define frailty as Tse et al. [5] in their systematic review and meta‐analysis for LVAD implantation—to non‐frail patients, frail patients had significantly longer time‐to‐extubation (mean difference: 45 ± 6 h) and hospital length of stay (mean difference: 2.9 ± 1.2 days; *p* = 0.001). It was also found that Frailty was not a predictor of inpatient or short‐term mortality (hazard ratio (HR): 1.22, 95% (CI): 0.66–2.26; *p* > 0.05) but predicted long‐term mortality (HR: 1.44, 95% CI: 1.15–1.80; *p* = 0.001) [4].

Considering the 2017 systematic review and the subsequent emergence of additional studies in the field, our objective is to reassess the outcomes of LVAD utilization in frail patients, taking into account the latest findings. Through this systematic review, we aim to corroborate the findings of earlier reviews and explore additional outcomes not covered in those studies. The primary objectives include a comparative analysis of short‐ and long‐term mortality, hospital length of stay, and duration of intubation between frail and non‐frail patients undergoing LVAD. Secondary outcomes encompass bleeding events, infection rates, and length of intubation.

## 2 Methodology

### 2.1 Literature Search Strategy

The meta‐analysis adhered to the guidelines outlined in the Preferred Reporting Items for Systematic Reviews and Meta‐Analyses (PRISMA) statement [7]. We conducted comprehensive searches in PubMed, Cochrane, EMBASE, MEDLINE, and Google Scholar from inception until September 2023.

The search terms used were: (“Left Ventricular Assist Device” OR “LVAD” OR “mechanical circulatory support” OR “Circulatory Support”) AND (“Frailty” OR “Frail” OR “Elderly” OR “Aging”). Further articles were identified through the use of the ‘related articles’ function on MEDLINE and a manual search of the references lists of articles found through the original search. Patient consent and IRB approval were not necessary in this study as no patients were deployed.

### 2.2 Study Inclusion and Exclusion Criteria

In this study, we included all studies that compared the outcomes of frail vs. non‐frail patients undergoing LVAD implantation. Studies were excluded if they fulfilled one of the following criteria: (1) studies using animal models, (2) small study population (< 10 people), (3) studies that do not have a comparison group, (4) inconsistencies in the data precluded valid extraction, or (5) case reports, abstracts from conferences, and preclinical studies. In line with the following criteria, two independent reviewers (C.C and A.A.R) selected articles for further assessment after title and abstract review. Disagreements between the two reviewers were resolved by a third independent reviewer (M.K.). Potentially eligible studies were then retrieved for full‐text assessment.

### 2.3 Data Extraction and Critical Appraisal

The entire content of the retrieved articles was meticulously examined and assessed by two authors, namely C.C. and A.A.R. The determination regarding the inclusion or exclusion of studies was reached through unanimous agreement between the aforementioned authors. In instances of disagreement, a third reviewer, M.K., assumed the responsibility of making the final decision. Employing a predetermined protocol, the extraction of the following data ensued: first author, study type and characteristics, number of patients, population demographics, in‐hospital mortality, overall mortality, length of intubation, hospital length of stay, major bleeding, infection, and re‐admission. The task of data extraction was carried out by two review authors, namely C.C. and A.A.R. The accuracy of the tabulated data was subsequently validated by a third author, M.K. Any potential disparities between the reviewers were amicably resolved through consensus.

### 2.4 Data Analysis

For dichotomous variables, encompassing outcomes such as short‐ and long‐term mortality, infection, and bleeding, we computed the odds ratio accompanied by a 95% confidence interval and corresponding p‐value. The Mantel–Haenszel random‐effects model was applied to aggregate the odds ratios derived from individual studies. In the context of continuous variables, specifically hospital length of stay and duration of intubation, we abstracted the mean and standard deviation. Statistical heterogeneity was assessed through the Chi‐squared test and *I* ^2^ test. Additionally, funnel plots were constructed to evaluate potential publication bias. Statistical significance was established at a two‐tailed *p* < 0.05. All analyses were executed using the “metafor” package within the R Statistical Software (version 4.0.2, Foundation for Statistical Computing, Vienna, Austria).

Individual patient data was reconstructed from published Kaplan–Meier graphs of all included studies using the “curve approach.” We followed the two‐stage method outlined by Liu et al., employing the R package “IPDfromKM” (version 1.2.3.0). Initially, raw coordinates (time, survival/event probability) were extracted from each subgroup within the Kaplan–Meier curves. Subsequently, these coordinates, along with the numbers at risk at specific time points, were used to generate individual patient data, indicating either time‐to‐event or time‐to‐last‐follow‐up for each patient. The resulting individual patient data was then combined to form a comprehensive dataset for each outcome.

### 2.5 Sensitivity Analysis

The impact of a single study on the overall effect of frail vs. non‐frail patients outcomes undergoing LVAD on the main outcome was assessed by sequentially removing one study (the “leave‐one‐out” method). This sensitivity analysis was carried out to test the consistency of these results to investigate if individual studies had an excessive impact on the results. Risk of bias analysis results are presented in Table 2.

## 3 Results

### 3.1 Description of Studies

**FIGURE 1:**
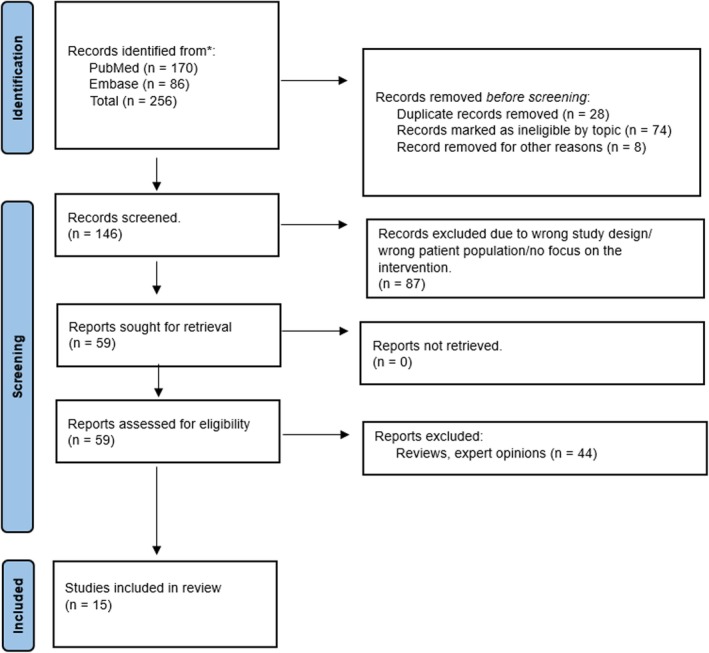
Flow diagram of the selected studies as created by Preferred Reporting Instructions for Systematic Reviews and Meta‐Analysis (PRISMA). [Color figure can be viewed at wileyonlinelibrary.com]

The literature search identified 185 articles. Of these, 71 relevant articles were read in full and assessed according to our inclusion and exclusion criteria. Following critical appraisal, a total of 15 studies incorporating a total of 3562 patients were included. The studies compared the outcomes of frail versus non‐frail patients undergoing LVAD implantation. Figure 1 illustrates the study selection process. All studies included were prospective or retrospective nonrandomized studies.

### 3.2 Baseline Characteristics

The baseline characteristics of the patients included in the studies are summarized in Table 1. Among these, 10 studies were prospective in design, and 6 were retrospective. There was a total of 3562 patients with a mean age of 59.7 years The average follow‐up period across these studies was 12 months. The assessment of frailty varied, with 7 out of the 15 studies utilizing Fried's score, 2 relying on clinical assessment, 2 employing sarcopenia as a criterion, and 2 using hand grip strength (HGS). One study utilized the deficit index, while another used a combination of cachexia and BMI reduction as measures of frailty. It is worth noting that the definition used for cachexia, involving > 10 kg weight loss or an absolute BMI < 20, is not universally accepted. Among the reported outcomes, long‐term mortality was the most studied (7 out of 15), followed by short‐term mortality (*n* = 4), length of intubation (*n* = 4), and hospital length of stay (*n* = 4). Additionally, other reported outcomes included infection (*n* = 2), post‐LVAD bleeding (*n* = 3), and readmission (*n* = 3).

**TABLE 1:** Summary of studies.

Study	Design	Country	Patients (frail vs. non frail)	Males (%)	Age, (mean)	INTERMACS score (mean) frail/non‐frail	Study period	Follow up period (months)	Definition of frailty	Primary outcome
Goldwater et al. [8]	PC	USA	20	65	69	N/A	02/2013−/11/2013	4	3/5 Fried's Score	Mortality measured as DAOH
Xu et al. [9]	RC	USA	115	N/A	N/A	N/A	N/A	6	Sarcopenia (Psoas Muscle Area)	HLOS, LOI
Manghelli et al. [10]	PC	USA	45 (31/14)	N/A	N/A	N/A	1/2013–9/2013	2	3/5 Fried Score	Length of ventilator, HLOS, mortality
Dunlay et al. [11]	PC	USA	99 (34/65)	81	65	4/7/4/7	01/2007–06/2012	23	Deficit index > 0.25	Mortality, HLOS in frail vs. non‐frail
Chung et al. [12]	PC	USA	72 (16/56)	65	59	N/A	10/2010–06/2013	5	HGS	Mortality, Bleeding risk, Infection
Yost et al. [13]	PC	USA	90 (45/45)	67	58	N/A	01/2005–01/2013	4 (days)	HGS	HLOS
Reeves et al. [14]	PC	USA	69 (54/15)	54	63	2.9/7/2.9/7	01/2012–12/2015	60	3/5 Fried's Score	Functional outcomes
Sundararajan et al. [15]	RC	N/A	154 (51/103)	68	N/A	N/A	01/2008–01/2015.	N/A	Cachexia > 10 kg weight loss or absolute BMI < 20	Mortality
Heberton et al. [16]	RC	USA	100 (32/68)	77	54	2/7/2/7	06/2008–08/2013	15	Sarcopenia (psoas muscle area)	Inpatient death, prolonged LOS of > 30 days
Cooper et al. [17]	RC	N/A	2469 (227/2242)	80	N/A	N/A	01/2012–03/2014	N/A	Clinician assessment (gait speed)	Mortality, infection, bleeding, rehospitalization
Jha et al. [18]	PC	USA	77 (18/59)	47	50	> 1/> 1	08/2013–08/2016	12	3/5 Fried's Score	Mortality, ILOS, HLOS
Jospeh et al. [19]	PC	USA	75 (44/31)	56	58	N/A	02/2013–04/2014	10	3/5 Fried's Score	Inpatient mortality, HLOS, extubation, and long‐term mortality
Muthiah et al. [20]	PC	Australia	96 (38/58)	18	56	2/7/2.3/7	01/2012–10/2019	12	3/5 Fried's Score	Mortality
Uzun et al. [21]	PC	Turkey	51 (28/23)	44	54	3/7/3/7	11/2016–11/2017	3	3/5 Fried's Score	Mortality, readmission, bleeding, renal dysfunction, VF/VT
Ajibawo et al. [22]	RC	USA	30 (12/18)	24	71	3/7/3/7	05/2018‐10/2020	12	Clinical Frailty Scale (CFS)	Mortality, HLOS, Intubation, Re‐admission

Abbreviations: DAOH, days out of hospital; HGS, hand grip strength; HLOS, hospital length of stay; ILOS, ICU length of stay; LOI, length of intubation in all studies the operation type was LVAD; PC, prosepective cohort; RC, retrospective cohort.

**TABLE 2:** Quality of risk of bias assessment using the Newcastle‐Ottawa Scale (NOS).

Study ID	Selection				Comparability	Outcome			Total (9)
	(a)	(b)	(c)	(d)	(e)	(f)	(g)	(h)	
1. Ajibawo et al. [22]	1	0	1	1	0	1	1	1	6
2. Uzun et al. [21]	1	0	1	1	0	1	1	1	6
3. Muthiah et al. [20]	1	0	1	1	1 (age)	1	1	1	7
4. Joseph et al. [19]	1	0	1	1	1 (LVEF)	1	1	1	7
5. Jha et al. [18]	1	0	1	1	2 (age, LVEF)	1	1	1	8
6. Cooper et al. [17]	1	0	1	0	0	1	1	1	5
7. Heberton et al. [16]	1	0	1	0	1 (age)	1	1	1	6
8. Sundararajan et al. [15]	1	0	1	0	1 (age)	1	1	0	5
9. Reeves et al. [14]	1	0	1	1	1 (age)	1	0	0	5
10. Yost et al. [13]	1	0	1	1	0	1	1	1	6
11. Chung et al. [12]	1	0	1	1	1 (age)	1	1	0	6
12. Dunlay et al. [11]	1	0	1	1	2 (age, LVEF)	1	1	1	8
13. Manghelli et al. [10]	1	0	1	1	0	1	1	0	5
14. Xu et al. [9]	1	0	1	1	0	1	1	1	6
15. Goldwater et al. [8]	1	0	1	1	0	1	1	1	6

*Note:* (a) Representativeness of the exposed cohort (1 point), (b) Selection of non‐exposed cohort (1 point), (c) ascertainment of exposure (1 point), (d) demonstration that the outcome of interest was not present at the start of the study (1 point), (e) comparability of cohorts (1–2 points), (f) assessment of the outcome (1 point), (g) follow‐up long enough for the outcomes to occur (1 point), (h) adequacy of follow‐up (1 point).

### 3.3 Quality Risk of Bias Assessment

In our meta‐analysis, we conducted a meticulous assessment of the included studies' quality using the Newcastle‐Ottawa Quality Assessment Scale (NOS). This assessment involved a structured scoring system designed to appraise three fundamental aspects: the selection of study participants, the comparability of study groups, and the quality of reported outcomes. We scrutinized various crucial attributes, including the representativeness of the cohort exposed to the factor of interest, the criteria used for selecting the nonexposed cohort, the confirmation of exposure status, the verification of the absence of the outcome of interest at the study's commencement, the comparability of cohorts concerning study design or analytical methods, the evaluation of study outcomes, the adequacy of follow‐up duration for the outcomes to manifest, and the completeness of cohort follow‐up. Studies meeting < 5/9 criteria were designated as of poor quality, 5–7/9 criteria were classified as moderate quality, and those satisfying ≥ 8/9 criteria were regarded as of good quality. In terms of study quality, four studies were of poor quality, nine studies were of moderate quality, and two were of good quality as per NOS. One notable concern revolved around the lack of matching between controls (non‐frail patients) and cases, particularly in terms of heart failure severity (LVEF), gender, and co‐morbidities. This discrepancy hinders the ability to objectively assert that the results are genuinely comparable or applicable.

### 3.4 Primary Outcomes

#### 3.4.1 Long Term Mortality

**FIGURE 2:**
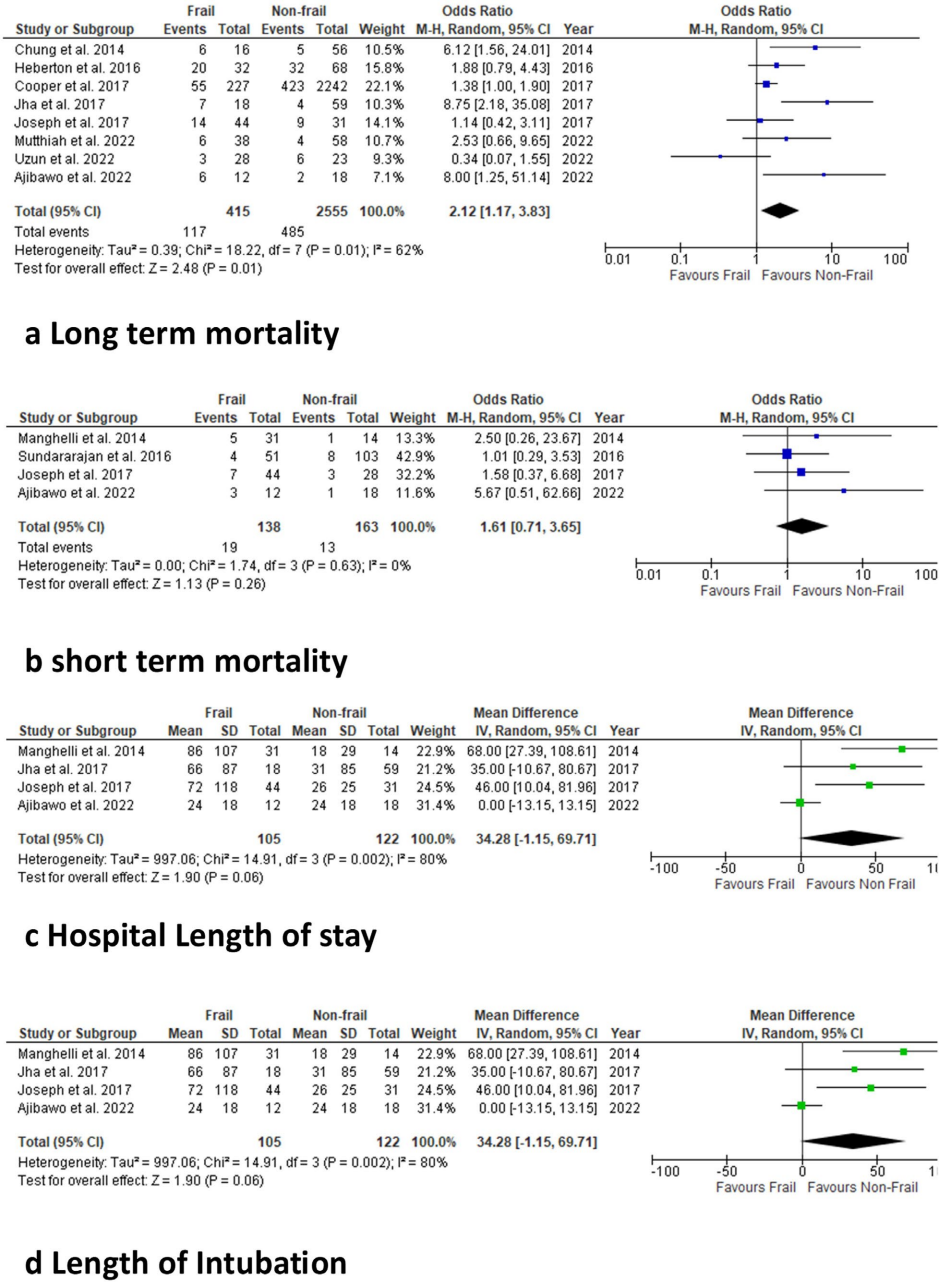
Meta‐analysis using forest plots for primary outcomes: (a) long‐ and (b) short‐term mortality, (c) hospital length of stay, and (d) length of intubation. [Color figure can be viewed at wileyonlinelibrary.com]

Frail patients undergoing LVAD were compared with non‐frail patients, with eight studies reporting on overall mortality outcomes postoperatively (Figure 2a). The overall long‐term mortality showed a statistically significant difference (random‐effects model: OR: 2.12; 95% CI: 1.17–3.83; *p* = 0.01). There was evidence of moderate heterogeneity among studies reporting on overall mortality (*I* ^2^ = 62%).

#### 3.4.2 Short Term Mortality

Frail patients undergoing LVAD were compared with non‐frail patients, with four studies reporting on short‐term mortality postoperatively (Figure 2b). Should you make figures separate or at least 2a, 2b, etc. for different outcomes? The overall OR for short‐term mortality showed no statistically significant difference between the two populations (random‐effects model: OR: 1.61; 95% CI: 0.71–3.65; *p* = 0.26). There was evidence of low heterogeneity among the selected studies (*I* ^2^ = 0%).

#### 3.4.3 Hospital Length of Stay

Frail patients undergoing LVAD were compared with non‐frail patients, with four studies reporting hospital length of stay (Figure 2c). The overall mean difference for hospital length of stay showed no statistically significant difference between the two populations (random‐effects model: mean difference: 34.28; 95% CI: −1.15 to 69.71, *p* = 0.75). There was evidence of high heterogeneity among the selected studies (*I* ^2^ = 80%).

#### 3.4.4 Length of Intubation

Frail patients undergoing LVAD were compared with non‐frail patients, with four studies reporting length of intubation (Figure 2d). The overall mean difference for length of intubation showed no statistically significant difference between the two populations (random‐effects model: mean difference: 34.28; 95% CI: −1.15 to 69.71; *p* = 0.06). There was evidence of high heterogeneity among the selected studies (*I* ^2^ = 80%).

### 3.5 Secondary Outcomes

#### 3.5.1 Bleeding

**FIGURE 3:**
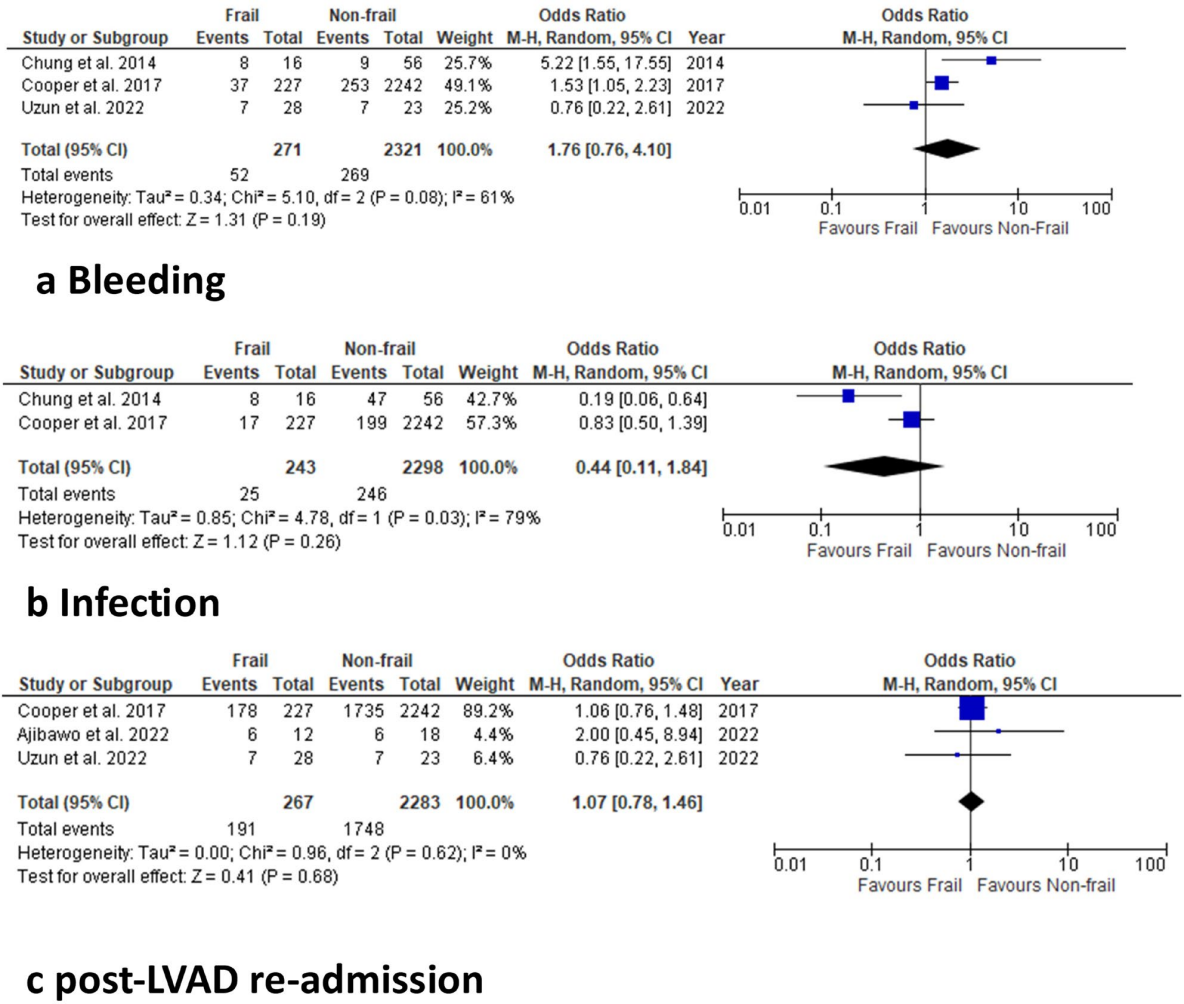
Meta‐analysis of secondary outcomes: (a)Post‐op re‐bleeding, (b) infection and (c) post‐LVAD readmission. [Color figure can be viewed at wileyonlinelibrary.com]

Frail patients undergoing LVAD were compared with non‐frail patients, with three studies reporting post‐operative bleeding (Figure 3a). The overall mean OR for bleeding showed no statistically significant difference between the two populations (random‐effects model: OR: 1.76; 95% CI: 0.76–4.10; *p* = 0.19). There was evidence of moderate heterogeneity among the selected studies (*I* ^2^ = 61%).

#### 3.5.2 Infection

Frail patients undergoing LVAD were compared with non‐frail patients, with two studies reporting post‐operative infection (Figure 3b). The overall mean OR for bleeding showed no statistically significant difference between the two populations (random‐effects model: OR: 0.44; 95% CI: 0.11–1.84; *p* = 0.26). There was evidence of high heterogeneity among the selected studies (*I* ^2^ = 79%).

#### 3.5.3 Post LVAD Re‐Admission

Frail patients undergoing LVAD were compared with non‐frail patients, with three studies reporting post‐LVAD re‐admission(Figure 3c). The overall mean OR for bleeding showed no statistically significant difference between the two populations (random‐effects model: OR: 1.07; 95% CI: 0.78–1.46; *p* = 0.68). There was evidence of low heterogeneity among the selected studies (*I* ^2^ = 0%).

### 3.6 Time to Event Meta‐Analysis

#### 3.6.1 Short Term Mortality Stratified by Frailty

**FIGURE 4:**
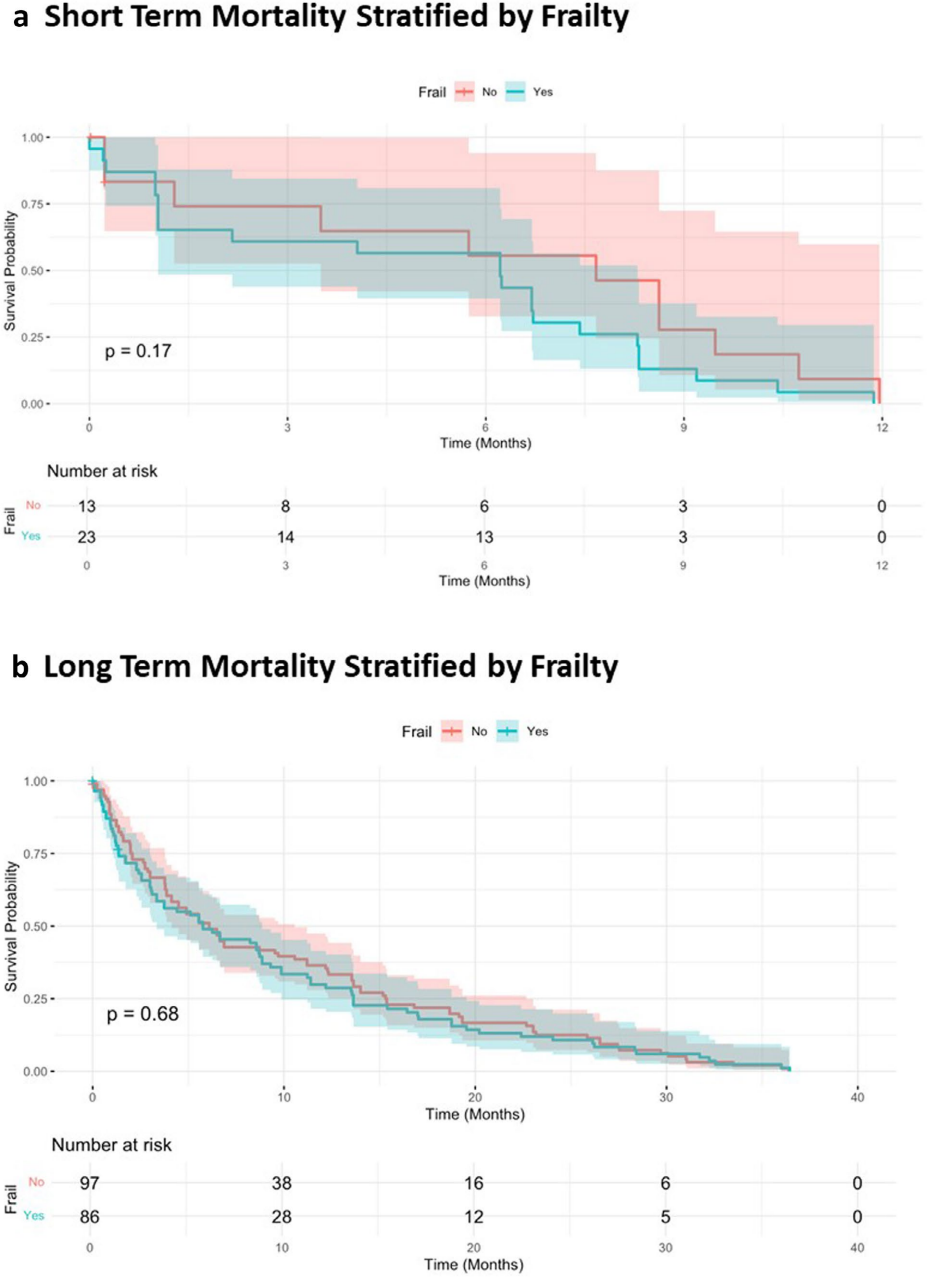
Kaplan–Meier curves show survival probabilities stratified by frailty for short‐term (a) (12 months) and long‐term (b) (40 months) periods. [Color figure can be viewed at wileyonlinelibrary.com]

The Kaplan–Meier curve (Figure 4a) for short‐term mortality (12 months) shows no statistically significant difference in survival between frail and non‐frail groups (*p* = 0.17). Frail individuals exhibit a slightly greater decline in survival probabilities during the first 3 months. However, by 12 months, survival probabilities are similar between the groups, with overlapping confidence intervals indicating comparable outcomes.

#### 3.6.2 Long Term Mortality Stratified by Frailty

The Kaplan–Meier curve for long‐term mortality (Figure 4b) (40 months) illustrates no statistically significant difference in survival between frail and non‐frail groups (*p* = 0.68). Both groups exhibit a gradual decline in survival probabilities over time, with consistent overlap in confidence intervals. The survival probabilities converge by the end of the study period, highlighting similar long‐term outcomes regardless of frailty status.

## 4 Discussion

### 4.1 Outcomes

In the 2017 study conducted by Tse et al., which entailed a systematic review and meta‐analysis comparing outcomes between frail and non‐frail patients, a total of 12 studies were incorporated [6]. Subsequent to the influx of new publications in the field, our meta‐analysis now encompasses data from 15 studies, involving a cohort of 3458 patients. Our primary outcome, focusing on long‐term mortality, reveals a statistically significant increase in long‐term mortality (> 12 months) among frail individuals undergoing (LVAD) placement when compared to their non‐frail counterparts. This finding aligns with both the previous systematic review and meta‐analysis and the majority of the reviewed literature, with the exception of the study by Uzun et al. [21] which suggests a favorable long‐term mortality in frail patients undergoing LVAD against non‐frail.

The analysis of short‐term mortality between frail and non‐frail patients undergoing LVAD reveals a non‐statistically significant association. Consistent with the prior meta‐analysis, the inclusion of the study by Ajibawo et al. did not significantly alter the overall OR in short‐term mortality. However, it is noteworthy that the study by Ajibawo et al. presents a considerably higher OR compared to previous studies, suggesting a need for further evidence regarding this outcome. Additionally, the study by Sundarajan et al. [15], which reports the least positive odds ratio, defines frailty as Cachexia (> 10 kg weight loss) or absolute BMI < 20. This implies that patients identified as frail by Sundarajan et al.'s criteria may not qualify as frail under Fried's Frailty Index or the Clinical Frailty Scale.

Regarding the duration of intubation, the mean difference between frail and non‐frail was similarly not statistically significant. This finding contradicts the results of the study by Tse et al. [5], where the mean difference was found to be statistically significant. The recent study by Ajibawo et al. [22], not included in the previous systematic review, reported no difference in the length of intubation between frail and non‐frail patients, thrusting the results into being statistically insignificant. However, employing the leave‐one‐out method by excluding the study of Ajibawo et al., the results become statistically significant. It is noteworthy that the other three studies [10, 18, 19] utilize the Fried's Frailty Index scale to define frailty, while the study by Ajibawo et al. employs the Clinical Frailty Scale.

### 4.2 Frailty

Frailty is a condition intricately associated with the aging process and delineated by Fried as a progressive decline in physiological system reserves and function, exhibiting a pronounced correlation with heightened risks of both hospitalization and mortality [23]. Previous meta‐analyses focusing on cardiac patients have underscored the escalated mortality risk associated with frailty in various cardiac interventions, including LVAD implantation, percutaneous coronary intervention, and transcatheter aortic valve implantation [4, 6, 24]. Our investigation contributes substantively to the scientific discourse by meticulously scrutinizing published data, concentrating on the nuanced interplay between frailty and clinical outcomes among advanced heart failure patients who have undergone LVAD implantation.

The absence of a universally accepted definition of frailty necessitates diverse methodologies for clinical management and research purposes, each replete with its own merits and drawbacks. Within gerontology and frailty assessment, Fried's phenotypic frailty model stands out as a principal tool. Originating from the Cardiovascular Health Study (CHS), this model encompasses five domains: grip strength, exhaustion, unintended weight loss, slow gait speed, and low physical activity [4]. Fried's model is the favored choice in the gerontology community for clinical applications due to its ease of application and precision. Predominantly, seven out of 15 studies on LVAD treatment utilized Fried's model for assessing frailty (references to the studies). The remaining eight studies employed surrogates or components of frailty scores, including sarcopenia, handgrip strength, and cachexia. In one study, a Deficit Index based on 31 deficits related to activities of daily living was employed. Notably, an absolute BMI < 20 does not universally denote cachexia; certain regions, such as parts of Asia, may exhibit such BMIs in elderly individuals without concomitant frailty considerations [15]. This observation is similarly applicable to sarcopenia—though associated with frailty, it does not necessarily equate to it.

### 4.3 Effect of Frailty on Outcomes

The augmented mortality observed in frail patients relative to their non‐frail counterparts is likely attributable to a confluence of factors. Frail individuals, characterized by diminished resilience to stressors, particularly evident in major surgical procedures such as LVAD implantation, may experience prolonged recovery periods, rendering them more susceptible to complications, including pneumonia or deep vein thrombosis. Postoperative complications documented in the literature encompass bleeding [25], myocardial infarction [26], and left ventricular thrombosis [27] The proinflammatory milieu associated with frailty may induce coagulopathy, heightening the risk of thrombotic events [28]. Additionally, heightened inflammatory activation may exacerbate local oxidative stress and create an arrhythmogenic substrate, predisposing individuals to ventricular arrhythmias and sudden death [5, 29].

Another pivotal consideration is the confounding influence of malnutrition in frail patients. Malnutrition, a prevalent condition in both frail individuals and those with sarcopenia [30], plays a significant role. Notably, suboptimal preoperative nutritional status correlates with unfavorable clinical outcomes in patients undergoing LVAD implantation [31, 32].

### 4.4 Limitations

One limitation identified is the variability in frailty indicators across different studies, leading to discrepancies in the definition of frailty. Frailty assessment methods within the selected studies include bioelectrical impedance analysis (BIA) and computed tomography (CT)‐based measurements of muscle areas relative to body surface area, such as the erector spinae muscle and the iliopsoas muscle. As a result, what qualifies as frailty in one study may not meet the criteria in another. It is important to note that this variability did not influence long‐term mortality outcomes.

In certain cases, the limited number of studies prevents the establishment of statistical significance, as observed in bleeding outcomes. This highlights the need for further reporting and investigation to comprehensively address these outcomes, which may stem from studies lacking adequate statistical power. Substantial heterogeneity was also noted, particularly in outcomes such as intubation duration and hospital length of stay, where study findings were inconsistent. To address this issue, we employed the leave‐one‐out method in our sensitivity analysis to mitigate heterogeneity and enhance the robustness of our results.

Finally, another limitation to consider is the variation in the types of LVADs used across different studies. Additionally, not all LVAD procedures were performed at the same center or by the same surgeon. This introduces potential variability due to differences in surgical technique and experiences, as well as the resources available at each center, which could influence outcomes and should be acknowledged as a potential downside.

### 4.5 Optimizing Outcomes in Frail Patients

The vulnerability of frail patients to unfavorable outcomes compared to their non‐frail counterparts is undeniable. To optimize outcomes for this population, the initial step is the identification of frail individuals. A variety of frailty screening instruments have been created for the general population, including the Clinical Frailty Scale, Frailty Phenotype, SHARE‐FI, and FRAIL [33, 34, 35].

Optimizing HF medication based on the latest guidelines is necessary to ensure that patients are adequately managed before undergoing LVAD surgery [36]. Starting treatment and modifying drug dosages (such as ACE inhibitors, beta‐blockers, diuretics, and SGLT2 inhibitors) can be particularly challenging for frail older adults. This challenge stems from an increased risk of adverse reactions due to comorbid conditions, polypharmacy, and potential drug interactions. Assessing the benefit–risk ratio can be difficult; however, tools like the STOPP and START criteria can help clinicians discern appropriate from potentially inappropriate medications for older adults [37]. Despite age variations, both randomized and observational studies involving elderly patients with HF demonstrate that these medications confer advantages [38, 39, 40, 41].

Exercise and nutrition may play a crucial role in the preoperative and postoperative management of frail patients undergoing LVAD implantation [42]. Endurance‐promoting activities, such as cycling and walking, have demonstrated improvements in physical capacity among heart failure (HF) patients [42]. Additionally, the combination of aerobic exercise and strength training has been shown to be effective in reducing muscle loss associated with heart failure [43]. However, the implications of these interventions for frail patients requiring LVAD and their impact on LVAD outcomes remain uncertain in the current literature. It is important to pair exercise regimens with nutritional strategies, as insufficient caloric intake has been associated with a decline in post‐discharge quality of life and an increased likelihood of readmissions in HF patients [44, 45]. Recommended strategies include dietary adjustments, the use of fortified nutrition, and oral protein‐energy supplementation when deemed necessary [35].

Malnutrition serves as a robust independent indicator associated with elevated perioperative mortality, increased morbidities, extended hospital stays, and higher rates of readmission. This, in turn, places a heightened burden on the healthcare system [46, 47]. The American Society for Enhanced Recovery and Perioperative Quality Initiative (ASER/POQI) succinctly outlined the existing challenges in perioperative nutrition screening and therapy. Accordingly, patients identified as being at high risk of malnutrition should be directed to a dietitian for a thorough nutritional assessment [48]. Avoidance of unnecessarily prolonged preoperative fasting is recommended. For patients with minimal risk of aspiration, unrestricted intake of solids should be permitted up to 6 h before anesthesia, and clear fluids up to 2 h before anesthesia [49].

## 5 Conclusion

Consistent with earlier observations, it is evident that long‐term mortality is more unfavorable in frail patients compared to non‐frail individuals undergoing (LVAD). Additionally, findings related to the length of intubation align with previous studies.

Notably, there is no indication that frailty influences infection rates, bleeding, or re‐admission post LVAD, based on the available data. However, the insufficient number of studies reporting on these outcomes and the underpowered nature of existing studies underscore the necessity for additional evidence to draw more conclusive findings. Recognizing frailty as a key risk factor for mortality can enhance risk stratification and inform candidacy for durable LVADs, while also guiding targeted interventions to address this occasionally modifiable clinical condition.

## Author Contributions

Christos Costa, Arian Arjomandi Rad, Yi Ting Yu, Nithiananthan Mayooran, and Marinos Koulouroudias contributed to the conception of the article and data collection, original writing, visualisation, revision and final approval. Arian Arjomandi Rad and Yi Ting Yu were responsible for the data analysis and visualization. Andrew Xanthopoulos, Robert Vardanyan, Gustavo Antonio Guida, Lydia Wilkinson, Jan Schmitto, Arjang Ruhparwar, Alina Zubarevich, Alexander Weymann, Peyman Sardari Nia, Antonios Kourliouros and Thanos Athanasiou contributed to the writing, revision of the article, and final approval of the manuscript. Peyman Sardari Nia, Antonios Kourliouros, Thanos Athanasiou supervision. All authors approved the article.

## Conflicts of Interest

The authors declare no conflicts of interest.
